# *In vivo* formaldehyde cross-linking: it is time for black box analysis

**DOI:** 10.1093/bfgp/elu037

**Published:** 2014-09-19

**Authors:** Alexey Gavrilov, Sergey V. Razin, Giacomo Cavalli

**Keywords:** formaldehyde cross-linking, chromatin, 3D genome organization, ChIP, 3C

## Abstract

Formaldehyde cross-linking is an important component of many technologies, including chromatin immunoprecipitation and chromosome conformation capture. The procedure remains empirical and poorly characterized, however, despite a long history of its use in research. Little is known about the specificity of *in vivo* cross-linking, its efficiency and chemical adducts induced by the procedure. It is time to search this black box.

We think it is urgent to draw attention to the uncertainty introduced in results obtained by ChIP and other formaldehyde fixation-based approaches by the fact the cross-linking efficiency of various proteins to DNA and to each other is drastically different and, in the case of *in vivo* cross-linking, may depend on local conditions within different cellular compartments.

Current chromatin research is characterized by the fast accumulation of genome-wide data on the distribution of various regulatory proteins along chromosomes. These data are easily accessible through different databases, and much effort has been made to pour more and more data into the pot. Surprisingly, however, not many scientists are concerned about the validity of the chromatin immunoprecipitation (ChIP) approach. The ChIP procedure was developed >15 years ago [1] and, essentially, the original protocol is still used without paying much attention to its inherent problems, even though it has long been felt that ‘the devil is in the ChIP details’. The most problematic step is formaldehyde fixation. It is commonly believed that formaldehyde can fix any DNA–protein complex. However, this assumption is far from being universally verified. For example, the lac repressor cannot be fixed to DNA by formaldehyde, even though its DNA-binding domain contains a number of basic amino acid residues [2]. The same was reported for NF-κB [3]. To specialists in the field, there are chromatin components that are proverbially difficult to cross-link, and specific protocols have been elaborated to solve this problem in some individual cases, mainly in an empiric way (see for instance [4]). It was established that there is a temporal threshold for cross-linking reactions such that once the residence time of a protein drops to <5 s, it becomes ‘invisible’ to formaldehyde cross-linking [5]. The formaldehyde fixation procedure remains in fact empirical, and little is known about the specificity of *in vivo* cross-linking, its efficiency and the chemical adducts induced by this procedure. Therefore, scientists performing cross-linking experiments are actually flying blind, and this can cause major problems in data interpretation [6, 7].

In recent years, methods (3C, 4C, Hi-C, ChIA-PET, etc.) based on the chromosome conformation capture (3C) procedure [8] have been widely used to study promoter–enhancer interactions and other questions related to the 3D architecture of the genome [9]. The 3C protocol is based on the assumption that DNA–protein complexes assembled in living cells can be fixed by formaldehyde, and then, after DNA cleavage by restriction enzymes, the complexes containing remote regulatory sequences linked by protein bridges can be solubilized and subjected to different treatments in solution. Recent studies from our groups have shown that this is not the case. Instead, formaldehyde fixation produces a rigid network of chromatin fibers that survives treatment with sodium dodecyl sulphate and restriction enzymes. Although this cross-linked chromatin network can be disrupted by sonication, many otherwise detectable contacts between DNA regulatory elements, such as promoters and enchancers of beta-globin genes, appear to be lost after such treatment [10–12]. These results argue that, in living cells, cross-linking of genomic elements via bridges made by regulatory proteins may be a relatively rare event in comparison with cross-linking of chromatin fibers via histones. This may reflect both infrequent juxtaposition of enhancers and promoters and inefficiency of formaldehyde cross-linking. Indeed, there are known examples demonstrating that enhancer–promoter interactions captured by 3C methods do not correlate with colocalization of these elements *in vivo* assayed by microscopy [13]. On the other hand, formaldehyde was reported to be inefficient for cross-linking of proteins that are not directly bound to DNA, such as transcriptional coactivators and corepressors [14, 15].

These observations prompt further questions: ‘are there differences between the efficiency of cross-linking in euchromatin and heterochromatin, and if so how do they correlate with genome-wide 3C data?’ For instance, Sanyal *et al.* reported higher 5C contact frequency in open chromatin regions [16], but it is unclear whether this result may be partly due to technical limits of 3C technology favoring cross-linking of open chromatin. In another study from the same laboratory, it was shown that in mitotic chromosomes both the large-scale spatial segregation and topologically associating domains were lost [17], but as of today, one cannot exclude the possibility that this pattern may be partly due to inefficient formaldehyde cross-linking of the highly condensed chromatin of mitotic chromosomes. On the other hand, the ability to detect 3C contacts appears to be dependent on the preservation of the architecture of unlysed nuclei [10]. As there is no nucleus in mitosis, it may be the absence of this architecture/nuclear compartments that may underlie the absence of 3C contacts in mitosis. Even more worrying results were obtained from the ChIP-seq analysis of distribution of the Silent information regulator (Sir) complex in *Saccharomyces cerevisiae*. The authors of this study discovered artifactual enrichment of multiple unrelated proteins, including the entire silencing complex, at highly expressed genes, calling into question the results of some previously published ChIP studies [6]. The observed phenomenon is most likely related to the existence of so-called high-occupancy target regions or ‘hotspots’ at which many DNA-binding proteins display a signal of enrichment despite the absence of an *in vitro* binding site in the underlying DNA sequence [18].

The chemistry of formaldehyde cross-linking is well known [1, 19], but it is the *in vivo* aspects of the technique that remain obscure. For example, formaldehyde fixation was reported to trigger DNA damage response and massive poly(ADP-ribosyl)ation of nuclear proteins, thus changing the chromatin composition and introducing bias in ChIP analysis [7]. The cross-links formed by formaldehyde treatment are fully and easily reversible by heating and a drop in pH, allowing for further analyses of both proteins and DNA. At the same time, the temperature and pH dependence of the cross-linking reaction raises a question of the stability of DNA–protein complexes obtained under different conditions in different applications. It is possible that minor variations in the conditions under which the cross-linking is performed can substantially affect the efficiency of cross-linking and/or the stability of the cross-linked products. This may apply not only to whole cells but also to local compartments within cells that may be embedded in different physicochemical microenvironments at the nanoscale of chromosome domains. It is thus not clear to what extent ChIP profiles reflect the distribution of the protein under study, and to what extent the local cross-linking conditions. The history of research shows remarkable examples of opposite conclusions made based on the results of cross-linking performed in slightly different ways [20].

We therefore wish to draw the attention of researchers to the necessity of reconsidering the basic steps of commonly used experimental protocols. As far as *in vivo* formaldehyde cross-linking is concerned, it is certainly time to upend some widely accepted assumptions. All evidence shows that formaldehyde cross-linking can no longer be used as if it was the molecular biology panacea. It is time to search this black box: investigate its *in vivo* molecular biology, its consequence on data collection in ChIP-seq and ‘C’ technologies, its limits, as well as to explore possible improvements and alternatives.

**Key points**

- Formaldehyde cross-linking is commonly used to probe chromatin structure but remains a poorly understood ‘black box’ technology.
- Cross-linking efficacy of different proteins to DNA and to each other varies widely.
- To interpret correctly the ChIP and 3C/Hi-C data, we definitely need to reinvestigate the limits and weaknesses of formaldehyde cross-linking procedure.

## FUNDING

The work of Alexey Gavrilov was supported by the Russian Science Foundation (14-14-01088). Giacomo Cavalli was supported by the ANR (iPolycomb), by the Foundation ARC and by the European Research Council (AdG N. 232947).

## References

[1] Orlando V, Strutt H, Paro R (1997). Analysis of chromatin structure by *in vivo* formaldehyde cross-linking. Methods.

[2] Solomon MJ, Varshavsky A (1985). Formaldehyde-mediated DNA-protein crosslinking: a probe for *in vivo* chromatin structures. Proc Natl Acad Sci USA.

[3] Nowak DE, Tian B, Brasier AR (2005). Two-step cross-linking method for identification of NF-kappaB gene network by chromatin immunoprecipitation. Biotechniques.

[4] Kurdistani SK, Robyr D, Tavazoie S (2002). Genome-wide binding map of the histone deacetylase Rpd3 in yeast. Nat Genet.

[5] Schmiedeberg L, Skene P, Deaton A (2009). A temporal threshold for formaldehyde crosslinking and fixation. PLoS One.

[6] Teytelman L, Thurtle DM, Rine J (2013). Highly expressed loci are vulnerable to misleading ChIP localization of multiple unrelated proteins. Proc Natl Acad Sci USA.

[7] Beneke S, Meyer K, Holtz A (2012). Chromatin composition is changed by poly(ADP-ribosyl)ation during chromatin immunoprecipitation. PLoS One.

[8] Dekker J, Rippe K, Dekker M (2002). Capturing chromosome conformation. Science.

[9] de Wit E, de Laat W (2011). A decade of 3C technologies: insights into nuclear organization. Genes Dev.

[10] Gavrilov AA, Gushchanskaya ES, Strelkova O (2013). Disclosure of a structural milieu for the proximity ligation reveals the elusive nature of an active chromatin hub. Nucleic Acids Res.

[11] Comet I, Schuettengruber B, Sexton T (2011). A chromatin insulator driving three-dimensional Polycomb response element (PRE) contacts and Polycomb association with the chromatin fiber. Proc Natl Acad Sci USA.

[12] Gavrilov AA, Chetverina HV, Chermnykh ES (2014). Quantitative analysis of genomic element interactions by molecular colony technique. Nucleic Acids Res.

[13] Williamson I, Eskeland R, Lettice LA (2012). Anterior-posterior differences in HoxD chromatin topology in limb development. Development.

[14] Zeng PY, Vakoc CR, Chen ZC (2006). *In vivo* dual cross-linking for identification of indirect DNA-associated proteins by chromatin immunoprecipitation. Biotechniques.

[15] Liang J, Lacroix L, Gamot A (2014). Chromatin immunoprecipitation indirect peaks highlight long-range interactions of insulator proteins and Pol II pausing. Mol Cell.

[16] Sanyal A, Lajoie BR, Jain G (2012). The long-range interaction landscape of gene promoters. Nature.

[17] Naumova N, Imakaev M, Fudenberg G (2013). Organization of the mitotic chromosome. Science.

[18] Ward LD, Wang J, Bussemaker HJ (2014). Characterizing a collective and dynamic component of chromatin immunoprecipitation enrichment profiles in yeast. BMC Genomics.

[19] Klockenbusch C, O'Hara JE, Kast J (2012). Advancing formaldehyde cross-linking towards quantitative proteomic applications. Anal Bioanal Chem.

[20] Nacheva GA, Guschin DY, Preobrazhenskaya OV (1989). Change in the pattern of histone binding to DNA upon transcriptional activation. Cell.

